# Isolation, characterization and application of a lytic phage vB_VspM_VS1 against *Vibrio splendidus* biofilm

**DOI:** 10.1371/journal.pone.0289895

**Published:** 2023-09-01

**Authors:** Xuemei Duan, Liming Jiang, Ming Guo, Chenghua Li

**Affiliations:** 1 State Key Laboratory for Quality and Safety of Agroproducts, Ningbo University, Ningbo, China; 2 Collaborative Innovation Center for Zhejiang Marine High-efficiency and Healthy Aquaculture, Ningbo University, Ningbo, China; 3 School of Medicine, Ningbo University, Ningbo, China; Universidad de Valparaiso, CHILE

## Abstract

*Vibrio splendidus* is a common pathogen in the ocean that infects *Apostichopus japonicus*, *Atlantic salmo*n and *Crassostrea gigas*, leading to a variety of diseases. In this study, a virulent phage vB_VspM_VS1, which infects *V*. *splendidus*, was isolated from aquaculture ponds in Dalian, China, and it belongs to the family *Straboviridae* in the order *Caudoviricetes*. vB_VspM_VS1 had an adsorption rate of 96% in 15 min, a latent period of 65 min, and a burst size of 140 ± 6 PFU/cell. The complete genome of phage vB_VspM_VS1 consists of a linear double-stranded DNA that is 248,270 bp in length with an average G + C content of 42.5% and 389 putative protein-coding genes; 116 genes have known functions. There are 4 tail fiber genes in the positive and negative strands of the phage vB_VspM_VS1 genome. The protein domain of the phage vB_VspM_VS1 tail fibers was obtained from the Protein Data Bank and the SMART (http://smart.embl.de) database. Bacterial challenge tests revealed that the growth of *V*. *splendidus* HS0 was apparently inhibited (OD_600_ < 0.01) in 12 h at an MOI of 10. In against biofilms, we also showed that the OD_570_ value of the vB_VspM_VS1-treated group (MOI = 1) decreased significantly to 0.04 ± 0.01 compared with that of the control group (0.48 ± 0.08) at 24 h. This study characterizes the genome of the phage vB_VspM_VS1 that infects the pathogenic bacterium *V*. *splendidus* of *A*. *japonicus*.

## Introduction

*Vibrio splendidus* is a gram-negative ubiquitous inhabitant pathogen of brackish water and marine environments that is associated with epizootics, including *Apostichopus japonicus*, crustaceans and fishes [1–3]. However, at present, there is no good drug treatment for *V*. *splendidus*. Li et al. reported that persister cells of *V*. *splendidus* were formed in culture with 10 minimum inhibitory concentration (MIC) of tetracycline and ciprofloxacin, persister cells are difficult to eradicate by antibiotic [4]. In addition, antibiotic-resistant bacteria are frequently found in aquaculture [5, 6]. Therefore, it is necessary to develop the application potential of phages against aquaculture pathogens.

Phages are viruses that can infect bacteria, spirochetes and actinomycetes [7]; they are highly host-specific and do not infect humans or animals. They parasitize only host cells that are susceptible to infection, so they can be used as ideal antibacterial biological agents to replace antibiotics [8]. With the use of antibiotics, the problem of resistant bacteria is becoming increasingly serious; therefore, there is an urgent need for phage-derived products (endolysins) and phages to replace antibiotic drugs [9, 10]. A number of phages PVS-1, PVS-2, PVS-3 and vB_VspP_pVa5, that infect *V*. *splendidus* have been reported, and their possess the efficacy to inactivate *V*. *splendidus*, the cultures infected with PVS-1, PVS-2, PVS-3 and vB_VspP_pVa5 at a MOI of 10 started to show significant decreases in optical density (OD_600_) compared with the control culture at 3 h after infection (*P* < 0.05) [11]. However, information on the genomes of *V*. *splendidus* phages has not been reported. Recently, many studies have reported that phages are effective in the treatment of *V*. *splendidus* or other bacteria important for aquaculture, Wu et al. reported that novel *Vibrio harveyi* phage vB_VhaS_PcB-1G exhibited a broad host range against *V*. *harveyi* (33/54) and large burst sizes (210 PFU/cell). Zhu et al. found that phage XC31 could kill *Vibrio mediterranei* 117-T6 and significantly decrease the *Vibrio* density in the infection system (P < 0.05). Rasmussen et al. found that combining phage KVP40 and probiont *Phaeobacter inhibens* DSM17395 resulted in a lower level of fish pathogenic *Vibrio anguillarum* than using the probiont alone [12–14].

Biofilms are highly resistant to antibiotics, desiccation, heat and acidic conditions [15]. Many bacteria in biofilms are approximately 10 to 1000 times less susceptible to antibiotics than culture bacteria for the reason of the extracellular polymeric substances of the biofilm prevent contact with antibiotics [16]. This makes the complete elimination of biofilms in the food industry, the clinic nearly and animal husbandry impossible [17].

Phages show high strain specificity to host bacteria, moreover, host bacteria are highly adaptable to phages. In a phage-host arms race, phage mutation occurs while the phage is infecting resistant bacteria [18–20]. Phage cocktails are effective against pathogenic bacteria that mutate and adapt. Hence, the continued screening of new phages is of great significance. Sequence analysis of the phage genome provides important resources for the development and utilization of phages. In this study, we identified a phage vB_VspM_VS1 that infects *V*. *splendidus*, and characterized its genome.

## Materials and methods

### Bacterial strains and growth conditions

Wild-type *V*. *splendidus* HS0 was isolated from diseased *A*. *japonicus* and stored in glycerol at -80 °C at Ningbo University, China, and used as the host bacterium. *V*. *splendidus* was grown aerobically on 2216E plates or in 2216E broth (Difco, Detroit, MI, USA), and incubated at 28 °C [21]. Soft top agar containing 0.5% (w/w) agar in 2216E broth was used for phage plaque confirmation, and 2216E agar plates containing 1.8% (w/w) agar were used for bacterial growth. *V*. *splendidus* HS0 was stored at −80 °C in 20% (v/v) glycerol.

### Phage isolation and purification

*V*. *splendidus* phage was isolated from *A*. *japonicus* breeding pond silt in Dalian, China. The propagation method was modified from Park [22]. Briefly, 5 g of a *A*. *japonicus* breeding pond silt sample was mixed with 15 mL phosphate buffer solution in a 50 mL centrifuge tube and incubated with shaking at 180 rpm for 1 h at room temperature. Then, the sewage sample was centrifuged at 6,000 × g for 10 min, and the supernatants were filtered with a 0.22 μm filter membrane. After filtration, 10 mL of each filtrate was inoculated onto log phase-grown *V*. *splendidus* in 40 mL of 2216E culture broth and incubated for 48 h. Then, the culture was centrifuged at 6 000 × g for 10 min, and the supernatant was filtered with a 0.22 μm filter membrane. The filtrate was serially diluted 10 times, mixed with 5 mL molten 0.5% 2216E soft agar containing *V*. *splendidus* HS0 (2 × 10^8^ cfu/mL), and immediately added to a 2216E plate. The growth of overnight cultures and plaque formation were observed after 24 h. A single phage plaque was selected for phage purification, and the process was repeated three times.

### Growth curve and adsorption rate of the isolated phage

To measure the adsorption rate, the co-culture of phage vB_VspM_VS1 (1 × 10^8^ pfu/mL) and logarithmic phase *V*. *splendidus* HS0 were mixed at an multiple of infection (MOI) of 1 and cultured at 28 °C; the phage titer was measured after 0, 5, 10, 15, 20, 25 and 30 min. The one-step growth curve of phage vB_VspM_VS1 was carried out as follows. Briefly, 10 mL of exponential phase *V*. *splendidus* HS0 culture was harvested by centrifugation (5,000 g, 4 min, 28 °C), and the pellet was resuspended in 20 mL of fresh 2216E to obtain an OD_600_ of 1.0. Next, 20 mL of phage vB_VspM_VS1 was added to reach an MOI of 1 and allowed to adsorb for 10 min at 28 °C. The mixture was centrifuged at 5,000 × g for 4 min at 28 °C, and the pellet was resuspended in 10 mL of fresh 2216E. Samples were taken every 10 min for 120 min, after which the supernatants were plated on 2216E agar to determine the phage titer.

### TEM and antibacterial analyses of the isolated phage

The morphology of the phage vB_VspM_VS1 particles was analyzed using transmission electron microscopy (TEM) mainly with the steps described below. Dilutions of the phage vB_VspM_VS1 stock (approximately 6×10^9^ pfu/mL) were deposited on carbon film and stained with 2% uranyl acetate. Phage vB_VspM_VS1 samples were observed using a Philips EM 300 electron microscope operated at an acceleration voltage of 120 kV at Ningbo University (Ningbo, China). Phage vB_VspM_VS1 was identified and classified according to the International Committee on Taxonomy of Viruses. Antibacterial assessment was performed by placing 6 mL diluted phage vB_VspM_VS1 (1 × 10^8^ pfu/mL) and 6 mL exponential phase *V*. *splendidus* HS0 culture (1 × 10^9^ cfu/mL) at an MOI of 0.1 in 150 mL 2216E culture, followed by incubation at 28 °C for 12 h; for the control, no phage was included. The absorbance was measured at a wavelength of 600 nm with a microplate reader. Phage concentration was determined on double layer 2216E agar at every 2 h. The experiment was repeated three times.

### Sequencing and analysis

Purified phage vB_VspM_VS1 was concentrated through a 10 kDa filter and treated with DNase and RNase at 37 °C for 1 h. A Takara Minibest Viral RNA/DNA Extraction Kit (Cat#9766) was used to extract phage vB_VspM_VS1 genomic DNA. Sequencing of the phage vB_VspM_VS1 genomic DNA was carried out using the Illumina HiSeq platform (Sangon Biotech, China) and assembled using SPAdes and FastQC assembler software. NCBI BLAST was used to compare sequences from multiple databases and open reading frames (ORFs), including TrEMBL, KOG, COG, CDD, NT, PFAM, SwissProt and NR, to obtain functional annotation information for the phage vB_VspM_VS1 gene protein sequences. The database VFDB (http://www.mgc.ac.cn/VFs/main.htm) was used to detect the virulence factors in the phage vB_VspM_VS1. The database ARG-ANNOT (http://backup.mediterranee-infection.com/article.php?laref=282&titre=arg-annot) was used to detect antimicrobial resistance genes in the phage vB_VspM_VS1. The genome similarity between phages was determined by BLASTN analysis via NCBI. The phylogenetic analysis of the phage vB_VspM_VS1 and related bacterial phage major capsid proteins amino acid sequences was performed using Molecular Evolutionary Genetics Analysis (MEGA7.0) software using the neighbor-joining method. The nodal reliability of the trees was assessed with bootstrapping using 500 pseudo replicates.

### *V*. *splendidus* phage vB_VspM_VS1 tail fibers structure domain

The protein crystal structures of the vB_VspM_VS1 tail fiber genes ORF42 (a), ORF61 (b), ORF64 (c) and ORF82 (d) of the *V*. *splendidus* phage were compared in the Protein Data Bank (PDB) database. a, b, c, and d of *V*. *splendidus* phage vB_VspM_VS1 tail fiber gene domains were predicted in the SMART (http://smart.embl.de) database.

### Determination of lytic capacity of phage vB_VspM_VS1 at different MOI

Four hundred microliter of the overnight culture was sub-cultured in 100 mL fresh 2216E liquid medium, added phage in culture according to the MOI = 0.1, 1 and 10. Sampling the culture of every 1 h (0–20 h) and use spectrophotometer to determination optical density (OD_600_). The effect of phage lytic capacity was examined in culture of *V*. *splendidus* by measuring the OD_600_ every hour at various MOI from 0.1 to 10. At the same time, Phages titers in the filtrates were determined by titration on the double-layer agar (0–12 h).

### Assessment of the effects of phage vB_VspM_VS1 on biofilm formation

To begin with, a 24-well cell slide was placed into a 12-well plate. The seed solution of *V*. *splendidus* was inoculated into 200 mL of 2216E culture at a concentration of 4‰. 1 mL of *V*. *splendidus* culture was inoculated into a 12-well plate. Phage vB_VspM_VS1 was added, with no addition used as a control group (the phage vB_VspM_VS1 was added at an MOI = 1), the cultures were incubated at 28 °C for 48 h and detected at 14, 24 and 48 h. Next, the recovered culture was washed three times with PBS buffer and fixed for 15 min with 99% methanol. Then, the methanol was discarded, and the *V*. *splendidus* cells were dried. A solution of 2% crystal violet was added and incubated with the cells for 15 min. The OD_570_ value was detected after rinses with water, and the results were obtained through epifluorescence microscopy. Three repeated tests were performed. The experiment was repeated three times.

## Results

### Characteristics and morphology of the isolated phage

**Fig 1:**
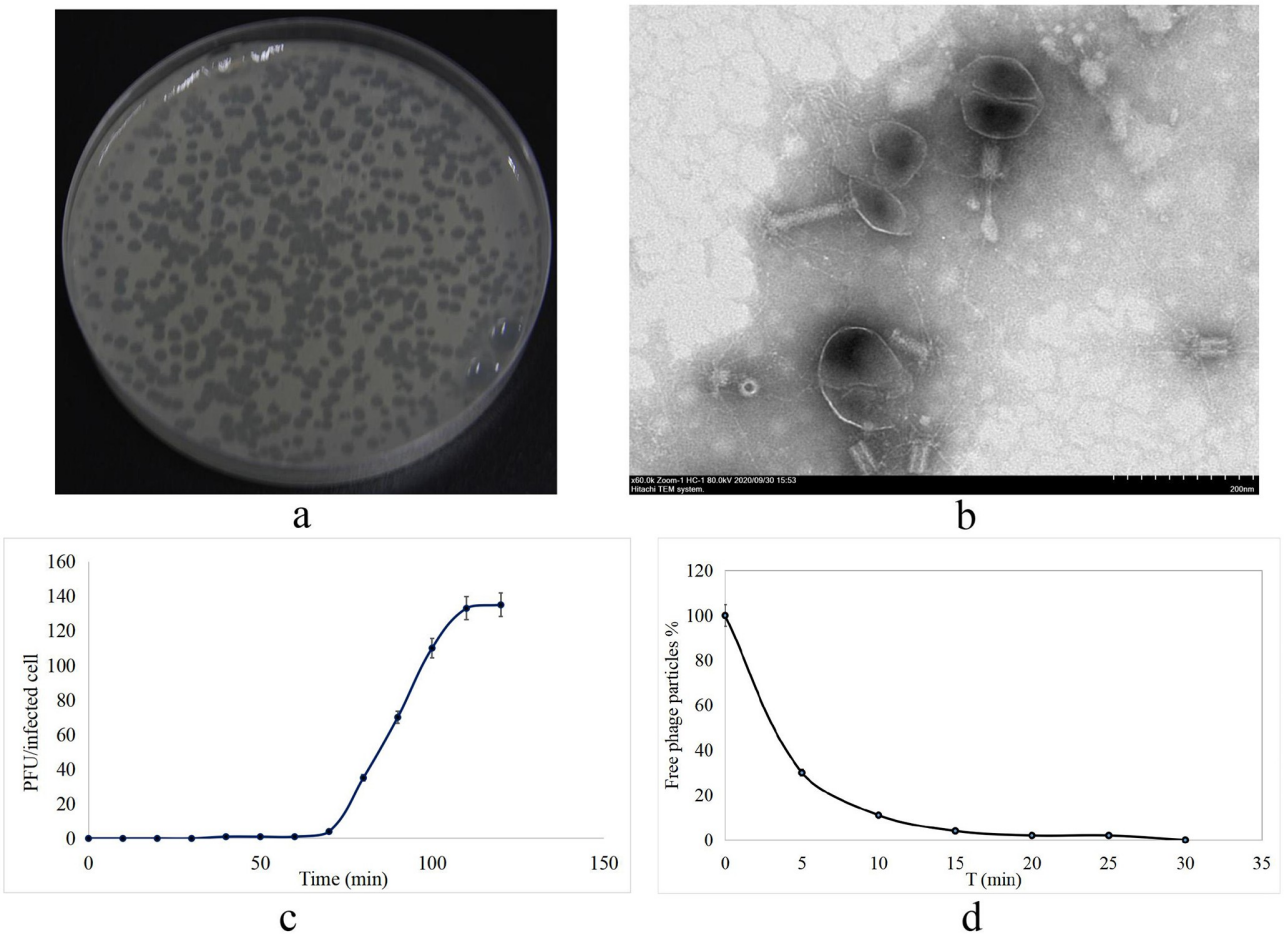
(a) Plaques formed by phage vB_VspM_VS1 on the host strain *V*. *splendidus* after overnight incubation at 28 °C. (b) Transmission electron micrograph showing that phage vB_VspM_VS1 belongs to the family *Myoviridae* and has a head of 119 × 143 ± 5 nm and a tail of 34 × 53 ± 5 nm. (c, d) Adsorption rate and population dynamics of phage vB_VspM_VS1 inoculated in *V*. *splendidus* culture. The values presented are means and standard deviations (SDs) of three independent biological repeats (n = 3).

Virulent *V*. *splendidus* phage vB_VspM_VS1 was isolated from a *A*. *japonicus* breeding pond silt in Dalian, China. The plaques of phage vB_VspM_VS1 were 3 mm in diameter after overnight incubation at 28 °C (Fig 1a). Negative staining of purified *V*. *splendidus* phage vB_VspM_VS1 was observed with an electron microscope. TEM showed that the phage vB_VspM_VS1 particles possessed an icosahedral head with a diameter of 143 ± 5 nm and a short tail with a length of 34 ± 3 nm (Fig 1b). The morphology and genomic data of phage vB_VspM_VS1 indicated that it belongs to the family *Straboviridae* [23]. A one-step growth curve of phage vB_VspM_VS1 was obtained by inoculation of *V*. *splendidus* HS0 at an MOI of 1 at 28 °C (Fig 1c). The latent period of phage vB_VspM_VS1 was 65 min, and the titers of phage vB_VspM_VS1 reached peaks very quickly in 2 h. In addition, the burst size of phage vB_VspM_VS1 was approximately 140 PFU/cell. After standing for 15 min at 28 °C, nearly 96% of the phage particles were adsorbed to the host bacterium *V*. *splendidus* HS0. After incubation for 30 min, almost all phages were adsorbed to the host bacterium *V*. *splendidus* HS0 (Fig 1d).

### Characterization of the genome

**Fig 2:**
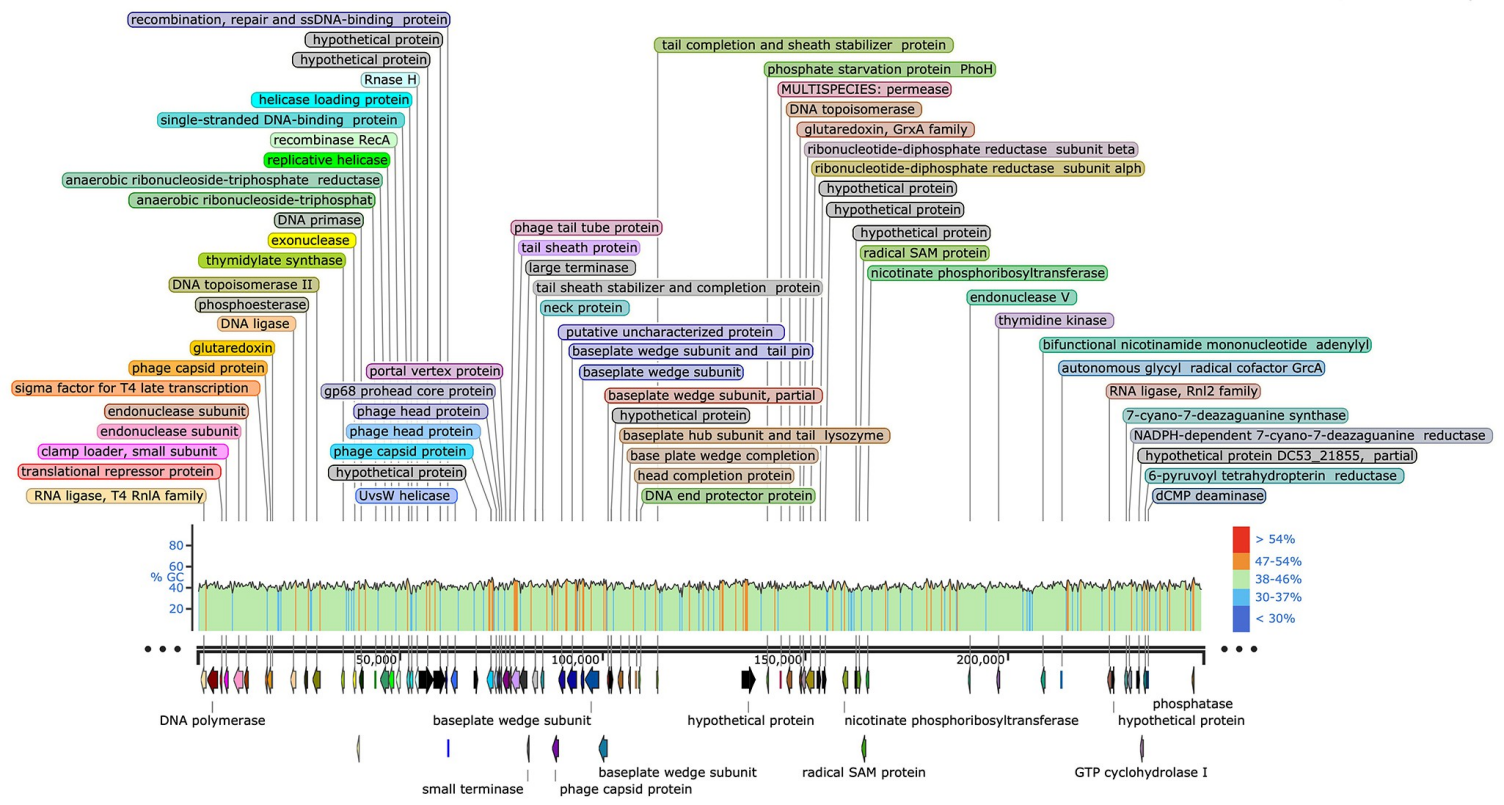
Line map of the vB_VspM_VS1 genome. In the vB_VspM_VS1 genome track, the arrows represent the ORFs and point in the direction of transcription. The colour intensity corresponds to G+C skew level.

To discern the novelty of vB_VspM_VS1 and its safety for biocontrol applications in the aquaculture industry, we characterized the phage genome. Phage vB_VspM_VS1 genomic DNA is a double-stranded with 248,270 base pairs in length. The G + C content of vB_VspM_VS1 was 42.5% (Fig 2). We identified 389 protein-coding genes [open reading frames (ORFs)] in the phage vB_VspM_VS1 genome, and 116 ORFs were annotated with specific functional genes (Table 1). The complete genomic sequences of phage vB_VspM_VS1 were deposited into the NCBI GenBank database (https://www.ncbi.nlm.nih.gov/nuccore) (GenBank accession number OK905446). Functionally annotated ORFs are shown in Table 1. Homologs of any integrases, virulence genes, transposases, and antibiotic resistance genes was absent from the genome; thus, we hypothesize that phage vB_VspM_VS1 would not form lysogens. Moreover, the phage vB_VspM_VS1 genome showed no homology with any published antimicrobial resistance genes (ARGs) or phage virulence factors. Additionally, related sequences were mostly found in *Vibrio* phage nt-1 (HQ317393.2). Furthermore, we analysed the genome similarity between phage vB_VspM_VS1 and nt-1 (HQ317393.2) and found that the Blastn sequence identity of vB_VspM_VS1 and phage nt-1 was 84.18% (S1 Fig in S1 File).

**Table 1:** A total of 116 ORFs were annotated as functional genes.

Feature ID	Start codon	Start	Stop	Length (bp)	Function
ORF1	ATG	1246	2391	1146	RNA ligase
ORF2	ATG	2752	5304	2553	DNA polymerase
ORF3	ATG	5948	6328	381	Translation repressor protein
ORF4	ATG	6363	6851	489	DNA replication
ORF5	ATG	6861	7817	957	DNA polymerase III
ORF6	ATG	7888	8553	666	Sliding clamp
ORF7	ATG	9315	11552	2238	Endonuclease subunit
ORF8	ATG	11775	12818	1044	Endonuclease subunit
ORF9	ATG	12815	13318	504	5’ nucleotidase
ORF10	ATG	13305	13769	465	Phage protein GP30.3
ORF11	ATG	13769	14125	357	Hypothetical protein
ORF12	ATG	17134	17646	513	RNA polymerase sigma factor
ORF13	ATG	17655	18551	897	Capsid vertex protein
ORF14	ATG	18610	18849	240	Arsenate reductase
ORF15	ATG	19007	19837	831	SprT-like family
ORF16	ATG	20607	22250	1644	ADP-ribosyltransferase
ORF17	ATG	23222	24562	1341	DNA ligase
ORF18	ATG	25050	25646	597	Hypothetical protein
ORF19	ATG	26776	27504	729	Phosphoesterase
ORF20	ATG	28875	30668	1794	DNA topoisomerase II
ORF21	TTG	30824	31564	741	NAD-dependent deacetylase
ORF22	ATG	33695	34456	762	Hypothetical protein
ORF23	ATG	34756	35073	318	Hypothetical protein
ORF24	ATG	35759	36658	900	Thymidylate synthase
ORF25	ATG	38687	39376	690	Exonuclease
ORF26	ATG	39635	40132	498	dUTPase
ORF27	ATG	40132	41190	1059	DNA primase
ORF28	ATG	41270	41749	480	Hypothetical protein
ORF29	ATG	41749	42573	825	DNA helicase UvsW
ORF30	GTG	43487	44011	525	Phosphoesterase
ORF31	GTG	43989	44456	468	4Fe-4S single cluster domain
ORF32	ATG	45541	47376	1836	Triphosphate reductase
ORF33	ATG	47613	48893	1281	41 helicase
ORF34	ATG	48950	49252	303	Phage head chap
ORF35	ATG	49311	50411	1101	RecA/RadA recombinase
ORF36	ATG	50479	51219	741	Caseinolytic protease
ORF37	ATG	51216	51767	552	Dihydrofolate reductase
ORF38	ATG	51818	52729	912	DNA binding protein
ORF39	ATG	52780	53430	651	Helicase loading protein
ORF40	ATG	53655	53927	273	Transcriptional regulator DsbA
ORF41	ATG	54010	54942	933	RnaseH
ORF42	ATG	55021	58761	3741	Long tail fiber
ORF43	ATG	61617	62030	414	ssDNA binding protein UvsY
ORF44	ATG	62027	62440	414	Yqey-like protein
ORF45	ATG	62531	62776	246	Protein of unknown function
ORF46	ATG	62926	64449	246	UvsW helicase
ORF47	ATG	70643	71722	1080	tRNA nucleotidyl transferase
ORF48	ATG	71774	73321	1548	Major capsid protein Gp23
ORF49	ATG	73392	74234	843	Prohead core protein
ORF50	ATG	74267	74908	642	Prohead core protein protease
ORF51	ATG	74911	75396	486	Prohead core protein
ORF52	ATG	75577	77124	1548	Phage T4-like Gp20
ORF53	ATG	77163	77663	501	Tail tube protein
ORF54	ATG	77715	79730	2016	Tail sheath protein
ORF55	ATG	79777	81576	1800	Large terminase protein
ORF56	ATG	81539	82081	543	DNA Packaging
ORF57	GTG	82795	84105	1311	Tail sheath stabilizer
ORF58	ATG	84115	84951	837	Virus neck protein
ORF59	ATG	84955	85878	924	Neck protein
ORF60	ATG	85891	87573	1683	Fibritin
ORF61	ATG	87870	89291	1422	Short tail fiber protein
ORF62	ATG	90826	91512	687	GP11 baseplate wedge protein
ORF63	ATG	91512	93758	2247	Baseplate and tail pin
ORF64	ATG	93768	94724	957	Baseplate tail fiber connector
ORF65	ATG	94781	95749	969	Baseplate wedge subunit
ORF66	ATG	95805	99302	3498	Baseplate wedge subunit
ORF67	ATG	99302	101260	1959	Baseplate wedge subunit
ORF68	ATG	101344	101763	420	Baseplate wedge subunit
ORF69	ATG	101948	102823	876	Band 7 or PHB domain
ORF70	ATG	103235	103531	297	PAAR motif
ORF71	ATG	103533	104015	483	Hypothetical protein
ORF72	ATG	104020	105237	1218	Tail lysozyme
ORF73	ATG	106510	107088	579	Baseplate wedge subunit
ORF74	ATG	107088	108233	1146	Tail-tube assembly protein
ORF75	ATG	108299	108754	456	Head completion protein
ORF76	ATG	109148	109744	597	DNA end protector protein
ORF77	ATG	110505	111353	849	Phage base plate protein
ORF78	ATG	111353	111520	168	Hypothetical protein
ORF79	ATG	113478	114008	531	Tail and sheath stabilizer protein
ORF80	ATG	114229	114867	639	Monophosphate kinase
ORF81	ATG	131366	134623	3258	Chaperone of endosialidase
ORF82	ATG	134694	137990	3297	Long tail fiber protein p37
ORF83	ATG	138964	139263	300	Anti-Sigma Factor A
ORF84	ATG	140539	141246	708	PhoH like protein
ORF85	ATG	144123	144377	255	Permease
ORF86	ATG	144413	145294	882	Organic radical enzymes
ORF87	ATG	145546	146832	1287	DNA topisomerase II
ORF88	ATG	148178	148747	570	Lytic Transglycosylase
ORF89	ATG	148821	149120	300	Glutaredoxin 1
ORF90	ATG	149122	150246	1125	Ribonucleotide reductase
ORF91	ATG	150256	152481	2226	Ribonucleoside reductase
ORF92	ATG	154537	155589	1053	Predicted Fe-S oxidoreductases
ORF93	ATG	159270	160763	1494	Phosphoribosyltransferase
ORF94	ATG	160831	162006	1176	LPS heptosyltransferase
ORF95	ATG	162986	164110	1125	Arylsulfatase regulator
ORF96	ATG	164103	165092	990	Radical SAM superfamily
ORF97	ATG	165095	165988	894	Phosphoribosyltransferase
ORF98	GTG	165960	166649	690	PP-loop superfamily ATPase
ORF99	ATG	176905	177729	825	CYTH-like superfamily
ORF100	ATG	183569	184243	675	Mononucleotide transporter
ORF101	ATG	185354	186334	981	AAA domain
ORF102	ATG	190447	190863	417	DNA glycosylase
ORF103	ATG	197488	198069	582	Thymidine kinase
ORF104	ATG	208309	209334	1026	ADP-ribose pyrophosphatase
ORF105	ATG	211854	213119	1266	AAA domain
ORF106	ATG	213142	213495	354	Glycyl radical enzyme
ORF107	ATG	213492	213866	375	Hypothetical protein
ORF108	ATG	224742	225728	987	RNA ligase
ORF109	ATG	226063	226572	510	Recombination endonuclease VII
ORF110	ATG	227217	227564	348	Chaperonin 10 Kd subunit
ORF111	ATG	228958	229674	717	Transcription regulator protein
ORF112	ATG	229729	230637	909	Deazaguanine reductase
ORF113	ATG	232779	233453	675	GTP cyclohydrolase I
ORF114	ATG	233521	234447	927	Tetrahydropterin reductase
ORF115	ATG	234502	234954	453	Deoxycytidylate deaminase
ORF116	ATG	246599	247516	918	Polynucleotide kinase

### Tail fiber gene domain and crystal structure information

**Fig 3:**
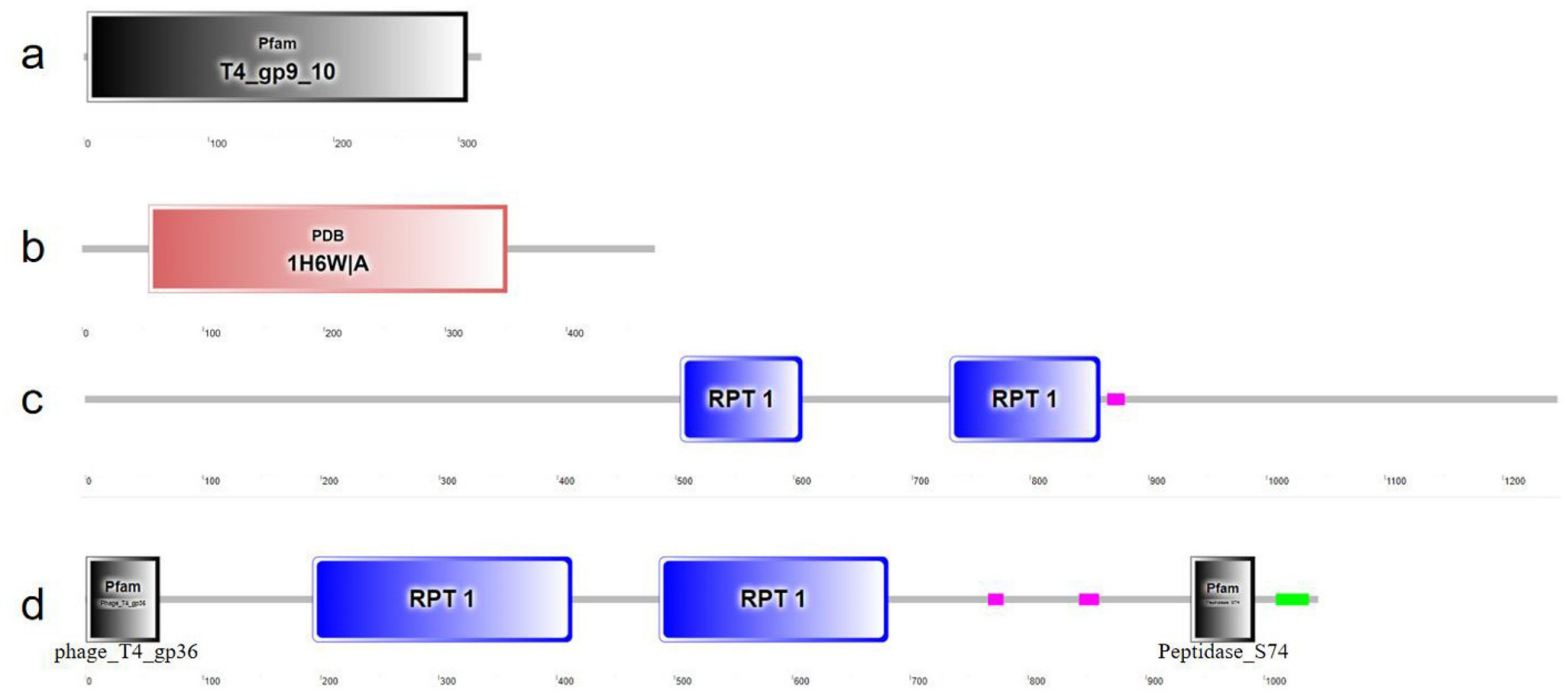
Schematic diagram domains of phage vB_VspM_VS1 tail fiber genes ORF42 (a), ORF61 (b), ORF64 (c) and ORF82 (d) obtained from the SMART (http://smart.embl.de) database.

Additionally, there were 4 tail fiber genes in the positive and negative strands of the phage vB_VspM_VS1 genome. The specific locations of phage vB_VspM_VS1 a, b, c and d tail fiber genes were 55021–58761 bp (ORF42), 87870–89291 bp (ORF61), 93768–94724 (ORF64) bp and 134694–137990 bp (ORF82), respectively. Protein domain analysis of the phage tail fiber proteins showed that tail fiber gene a likely generates a contractile tail, tail fiber gene b likely generates a short tail, tail fiber gene c likely generates a proximal long caudal fiber, and tail fiber gene d likely generates a long tail fiber. Prediction of the vB_VspM_VS1 tail fiber gene domains using the SMART (http://smart.embl.de) database showed that the tail fiber gene a, b, c-1, c-2, c-3, d-1, d-2, d-3, d-4, d-5, d-6 and d-7 domains consist of 305, 297, 104, 128, 16, 63, 221, 195, 14, 18, 55 and 29 amino acids, respectively (Fig 3).

### Phylogenetic analysis

**Fig 4:**
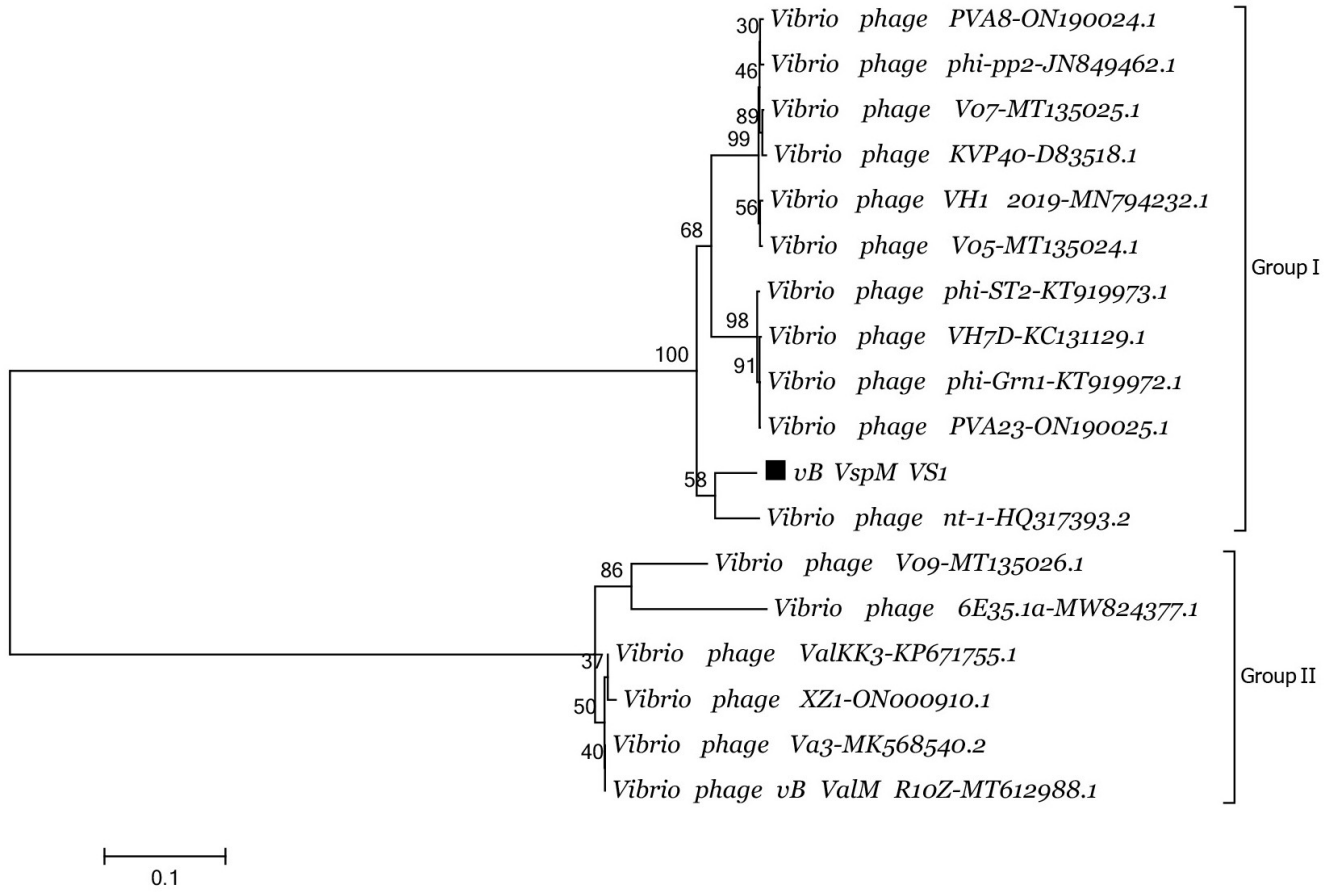
Neighbor-joining tree showing the relationship of various phages from the NCBI database to vB_VspM_VS1. The tree was drawn based on the major capsid amino acid protein of each phage. Bootstrap values were set for 500 repetitions. Phage vB_VspM_VS1 and nt-1 have very close evolutionary distance. Scale bar means distance ruler.

The amino acid sequences of *V*. *splendidus* and related bacterial phage major capsid proteins were obtained via the NCBI nucleotide database and compared (*Vibrio* phage nt-1, HQ317393.2; *Vibrio* phage V09, MT135026.1; *Vibrio* phage VH1_2019, MN794232.1; *Vibrio* phage V07, MT135025.1; *Vibrio* phage phi-ST2, KT919973.1, etc). Genome sequences from 17 other *Vibrio* group phages were obtained from the NCBI in GenBank format. A phylogenetic analysis of the major capsid proteins of all 18 phages (including vB_VspM_VS1) was conducted using the neighbor-joining method in MEGA7 and ClustalW (Fig 4). ClustalW was used to align the sequences, and MEGA7 was used to construct a neighbor-joining tree with 500 bootstrap replicates. This tree showed that phage vB_VspM_VS1 is highly homologous to the *Vibrio* phages nt-1, phi-ST2 and VH7D, which all belong to evolutionary Group I of a subclass of the *Schizotequatrovirus*, which share a common evolutionary branch (Fig 4).

### Antibacterial analyses of the isolated phage

**Fig 5:**
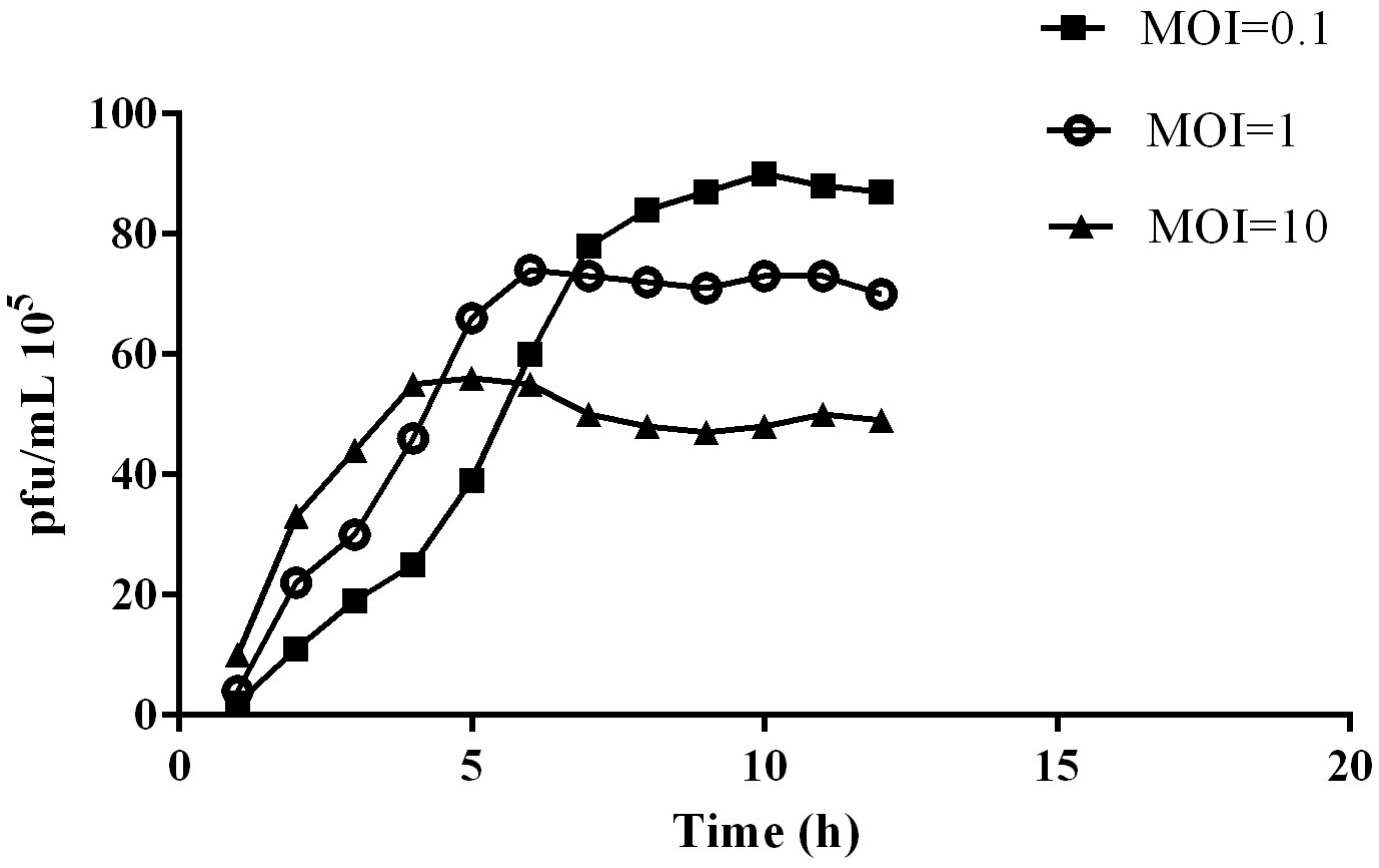
Corresponding abundances of pfu/mL were quantified by plaque assay over a 12 h period of incubation in *V*. *splendidus* cultures in the presence of phage vB_VspM_VS2 at a multiplicity of infection (MOI) of 0.1, 1 and 10 were measured at different incubation times, respectively.

**Fig 6:**
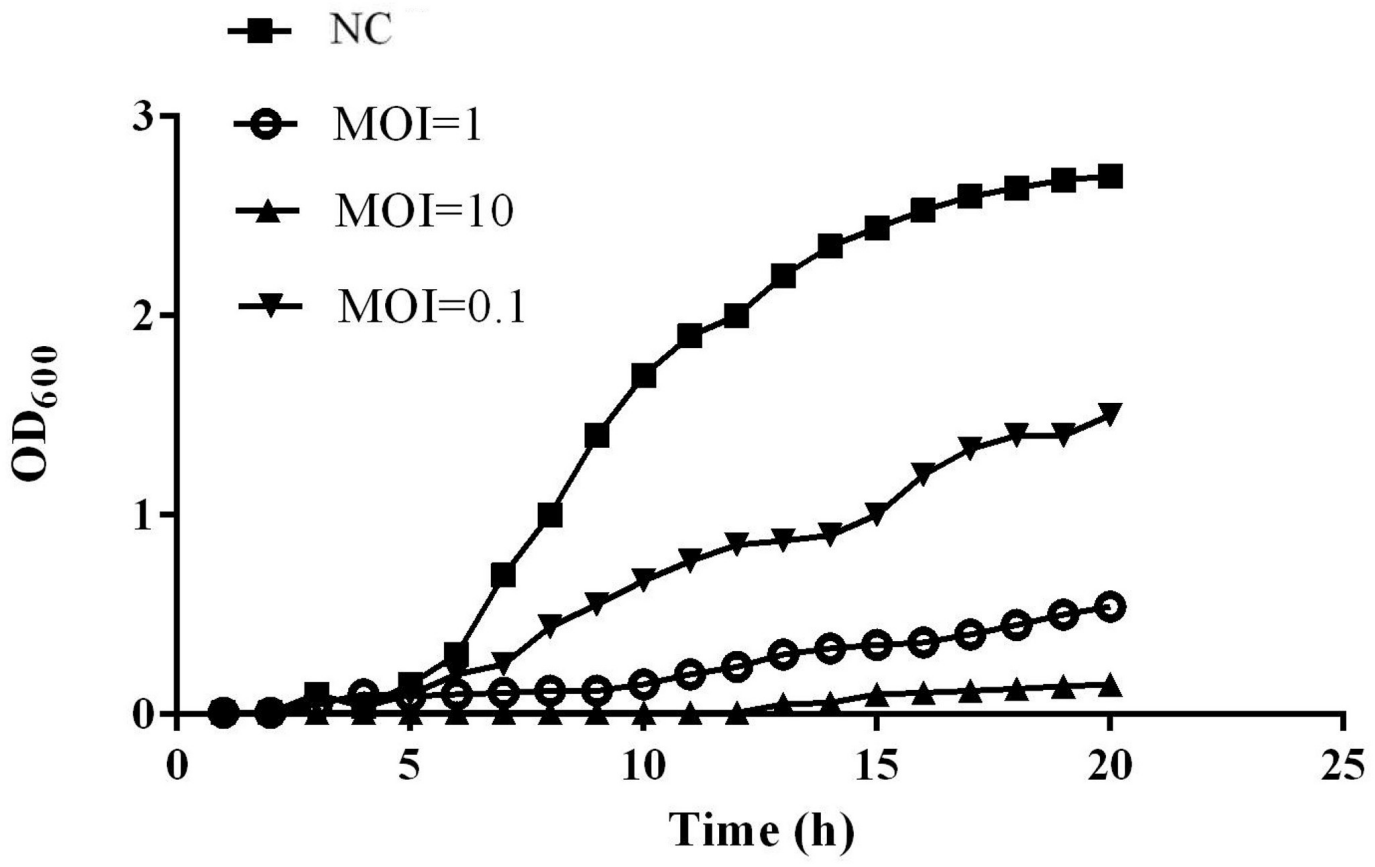
Comparison of the lytic ability of phage vB_VspM_VS1 using *V*. *splendidus* as the host at various MOI from 0.1 to 10 in 2216E broth.

Co-culture of phage vB_VspM_VS1 and *V*. *splendidus* showed increased eradication compared to *V*. *splendidus* HS0 culture according to the results of the microplate reader OD_600_. After culturing for 12 h, the OD_600_ value of *V*. *splendidus* HS0 was 2.15 at an inoculate rate of 4‰. There were significant differences seen in the co-culture of phage vB_VspM_VS1 and *V*. *splendidus* HS0 based on statistical data analysis software version 17.0 (ANOVA) (IBM, Chicago, USA). The OD_600_ value was significantly reduced to 0.023, and the bacteriostatic effect of phage vB_VspM_VS1 on *V*. *splendidus* HS0 culture was 93.5 times that of the control culture (Fig 6). The phage titer increased substantially within 4 h (Fig 5). We observed phage vB_VspM_VS1 propagation in the *V*. *splendidus* HS0 culture at different MOIs of 0.1, 1 and 10, whereas the phage production was ~2-fold lower in the MOI = 10 culture than in the MOI = 0.1 culture (Fig 5). The growth curves of *V*. *splendidus* HS0 indicated that the phage vB_VspM_VS1 had a better inhibitory effect at an MOI = 10, 1 and 0.1 (Fig 6). The combination of phage vB_VspM_VS1 and *V*. *splendidus* HS0 resulted in maximum lysis of the host strain at different MOIs (Fig 6). The growth of *V*. *splendidus* HS0 was apparently inhibited (OD_600nm_ < 0.01) in 12 h at an MOI of 10, whereas the OD_600nm_ started to increase after 3 h at MOI = 0.1 and 1.

### Anti-biofilm activity of phage vB_VspM_VS1

**Fig 7:**
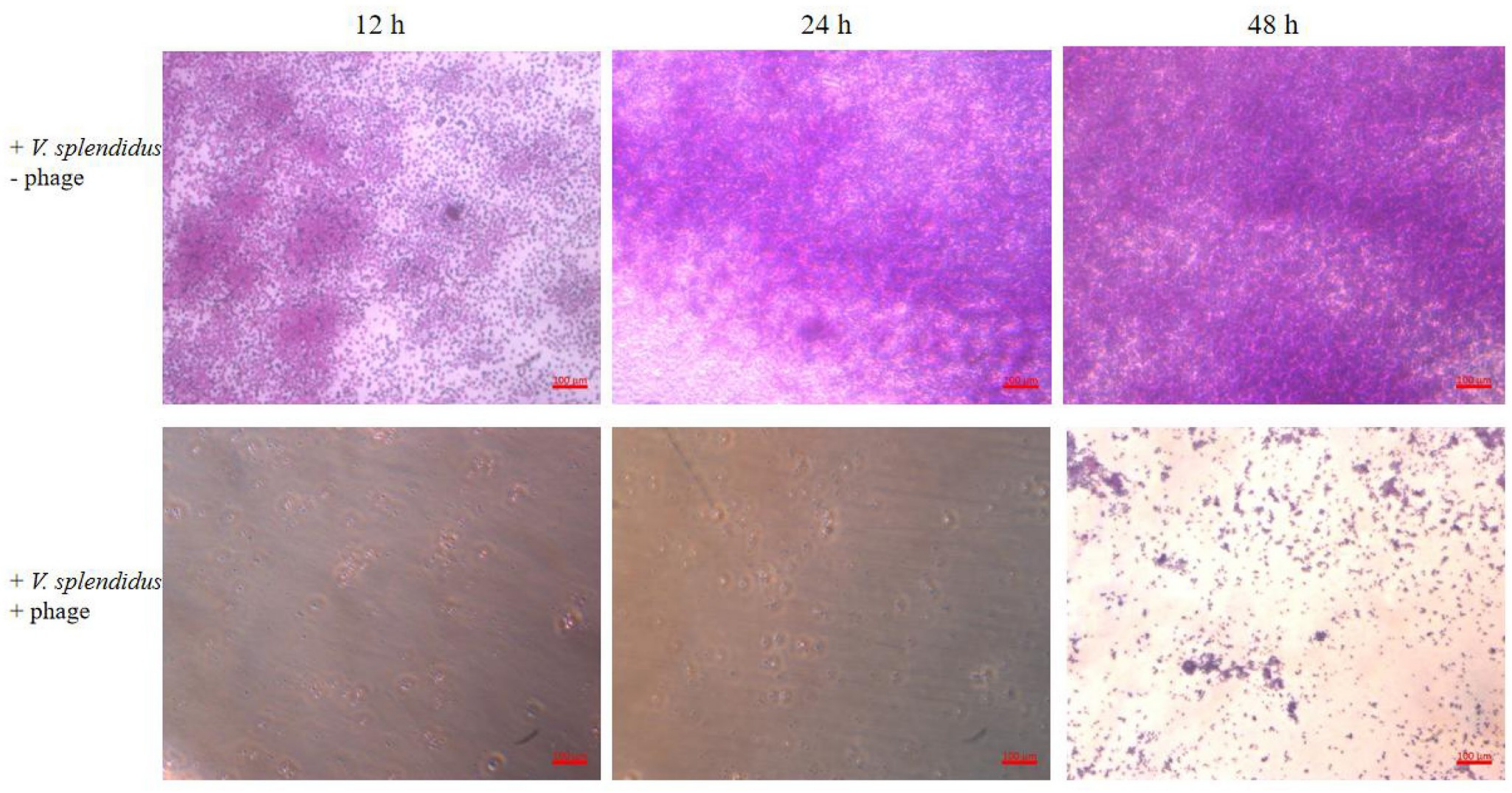
Biofilm formed by *V*. *splendidus* at 14 h, 26 h and 48 h. Biofilm formed after phage vB_VspM_VS1 treatment was observed by optical microscope. Phage vB_VspM_VS1 inhibited the formation of *V*. *splendidus* biofilm by MOI = 1 under the same conditions at 14 h, 26 h and 48 h.

**Fig 8:**
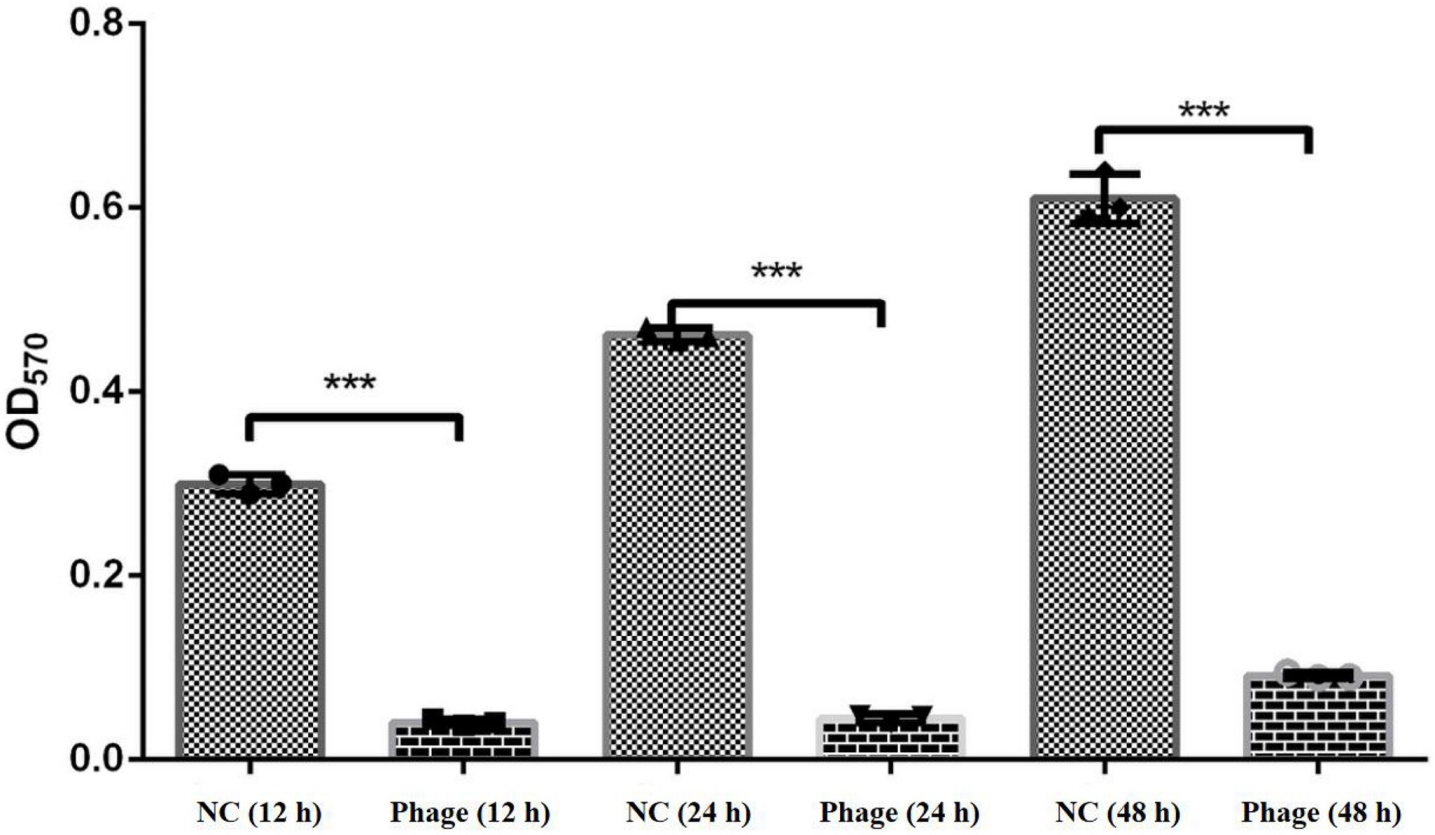
Effects on *V*. *splendidus* biofilm cultured in the presence of phage vB_VspM_VS1 on MOI = 1 for 12, 24 and 48 h (OD_570_).

Crystal violet staining experiments showed that *V*. *splendidus* had strong biofilm formation ability. To evaluate the ability of vB_VspM_VS1 to inhibit biofilm formation, the OD_570_ values of *V*. *splendidus* were measured at 14, 24 and 48 h, and it was found that the most biofilm was formed at 48 h. The effect of vB_VspM_VS1 (MOI = 1) on biofilm formation by *V*. *splendidus* was assessed using 96-well plates. The results showed that the OD_570_ values of the vB_VspM_VS1-treated group (MOI = 1) decreased significantly to 0.04 ± 0.01 compared with that of the control group (0.48 ± 0.08) at 24 h (Fig 8). This result suggested that vB_VspM_VS1 could prevent biofilm formation. This analysis demonstrated that phage vB_VspM_VS1 had a greater bactericidal effect (Figs 7 and 8).

## Discussion

The *V*. *splendidus* HS0 strain used in this study was isolated from *A*. *japonicus* suffering from skin ulceration syndrome at indoor farms at the Jinzhou Hatchery; unfortunately, it was resistant to ampicillin. The emergence of multidrug-resistant (MDR) strains urgently requires new measures to inhibit pathogens. With the overuse of antibiotics, multidrug-resistant bacteria and superbacteria have emerged and have led to public health problems [21]. The isolated *V*. *splendidus* phage vB_VspM_VS1 was identified as an ideal substitute for antibiotics due to its strong lytic activity. We have conducted two experiments on phage lytic activity against *V*. *splendidus*. When the MOI of phage vB_VspM_VS1 to *V*. *splendidus* was 0.01, phage vB_VspM_VS1 completely eliminated *V*. *splendidus* growth on the plate (S2 Fig in File). In another experiment, phage vB_VspM_VS1 completely inhibited the growth of *V*. *splendidus* for up to 12 h in the culture medium when the MOI was 10 (Fig 6). Sarropoulou et al. reported that *V*. *splendidus* phage vB_VspP_pVa5 absence of genes related to lysogeny along with the high efficacy observed during in vitro cell lysis trials indicated that the vB_VspP_pVa5 was a potential candidate for the biological control of *V*. *splendidus* in aquaculture [8]. Li et al. reported that during the 48-day cultivation time, the total *Vibrio* counts in phage treatment groups decreased in the coelomic fluids of *A*. *japonicus*, and at the end of 48-day cultivation time, the total weight gain of the *A*. *japonicus* in the phage treatment group increased significantly compared to the control group (*P < 0*.*01*) [24].

*V*. *splendidus* is one of the important pathogens to aquatic animal in mariculture. It has broad susceptible hosts, including shellfish, *A*. *japonicusins* [25], which has caused very large economic losses to the aquaculture industry. With the increase prevalence of bacteria antibiotic resistance in aquaculture, phage can be used to control bacterial disease as antibiotics are becoming an increasingly substandard option for disease control in aquaculture. Li et al. found that phage freeze-dried powder can significantly improve the survival rate of *A*. *japonicusins* seedlings that attributed to phage can obviously inhibit the growth of pathogenic *V*. *splendidus* [23]. This work tested the ability of the phage vB_VspM_VS1 to control *V*. *splendidus*. Phage vB_VspM_VS1 shown strong lytic activity against *V*. *splendidus* suggesting that the activity of the phage was specifific for *V*. *splendidus*. At the same time, this study annotated the phage genome in detail, which provides research materials for the development of genetic engineering technologies such as phage lyase and hydrolase.

The isolated *V*. *splendidus* phage vB_VspM_VS1 belongs to the family *Myoviridae*, and its genome size is 248270 bp. TEM revealed the phage vB_VspM_VS1 virions to have an icosahedral head of 119 × 143 ± 5 nm and a tail of 34 × 53 ± 5 nm in length (Fig 1b). In comparison, the *V*. *splendidus Myoviridae* phage PVS-1 virions have an icosahedral head of 45 ± 2 nm and a tail of 74 ± 1 nm in length, and phage PVS-2 virions have an icosahedral head of 95 ± 3 nm and a tail of 42 ± 2 nm in length. *V*. *splendidus Siphoviridae* phage PVS-3 virions have an icosahedral head of 47 ± 2 nm and a tail of 152 ± 2 nm in length [11, 23].

The structure and molecular specificity of phage tail fibers affects the adsorption, host range, gene expression and the development of cell fate of host bacteria [26]. The genomic characteristics of *V*. *splendidus* phage vB_VspM_VS1 revealed that vB_VspM_VS1 encodes 4 tail fiber genes in the positive and negative strands of the phage vB_VspM_VS1 genome. Phage tail fibers play an important role in infecting host bacteria. Kells et al. reported that T4 phage tail fibers irreversibly bind to the lipopolysaccharide core region of *E*. *coli* and are responsible for initial phage attachment [17]. Miernikiewicz et al. found that gp12, a phage T4 tail protein had the ability to bind to lipopolysaccharide on the bacterial surface [27]. Through natural evolution, structural modeling and site-directed mutagenesis, Yehl et al. determined that the HRDR region of the tail fiber of the T3 phage determines its host range [28].

In this study, a new *V*. *splendidus* phage, vB_VspM_VS1, was isolated and characterized. Through the comparison of key proteins of the major capsid and the large terminal subunit of phage vB_VspM_VS1, there no similar phages were found in the Blastn database. In addition, genome identity between phage vB_VspM_VS1 and nt-1 (HQ317393.2) was 84.18%. Here, we reported the genome characterization of a new *V*. *splendidus* phage. Thus, phage vB_VspM_VS1 has potential application value as an antimicrobial agent in the aquaculture industry.

## Supporting information

S1 File **S1 Fig**, Plot of comparative analysis of genome similarity between phage vB_VspM_VS1 and phage nt-1 (HQ317393.2). **S2 Fig**, The display diagram of the lytic activity of vB_VspM_VS1 for *V*. *splendidus*, shown that the infection of phage vB_VspM_VS1 with MOI = 0.01 (b) can cleave the entire pathogen on the plate, as a comparison, there were some bacteria can still grow on the plate with phage titer of 0.001 (a), indicating that the isolated phage has extremely strong lytic activity.
(DOC)
