# Supplementary material for: Isolation, characterization and application of a lytic phage vB_VspM_VS1 against Vibrio splendidus biofilm
Source: PLoS One. 2023 Sep 1;18(9):e0289895. doi: 10.1371/journal.pone.0289895 (PMC10473537; doi:10.1371/journal.pone.0289895)

# Supplementary Information

Isolation, characterization and application of a lytic phage vB_VspM_VS1 against *Vibrio splendidus* biofilm

Xuemei Duan^1,2^, Liming Jiang^1,2^*, Ming Guo^1,2^, Chenghua Li^1,2^

^1^ State Key Laboratory for Quality and Safety of Agroproducts, Ningbo University, Ningbo, 315211, China.

^2^ Collaborative Innovation Center for Zhejiang Marine High-efficiency and Healthy Aquaculture, Ningbo University, Ningbo, 315211, China.

^*^Correspondence and requests for materials should be addressed to LM. Jiang ([jiangliming@nbu.edu.cn)](mailto:jiangliming@nbu.edu.cn)).

**Figure S1.** Plot of comparative analysis of genome similarity between phage vB_VspM_VS1 and phage nt-1 (HQ317393.2).

Note. This dot matrix view shows regions of similarity based upon the BLAST results. The query sequence is represented on the X-axis and the numbers represent the bases/residues of the query. The subject is represented on the Y-axis and again the numbers represent the bases/residues of the subject. Alignments are shown in the plot as lines. Plus strand and protein matches are slanted from the bottom left to the upper right corner, minus strand matches are slanted from the upper left to the lower right. The number of lines shown in the plot is the same as the number of alignments found by BLAST.


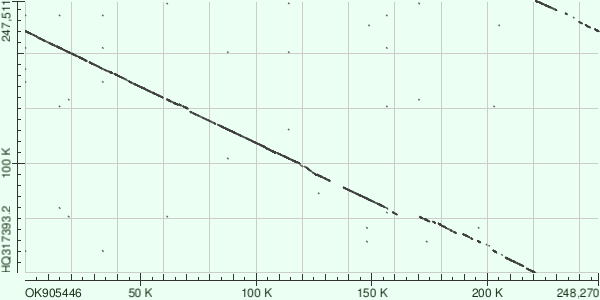


**Figure S2.** The display diagram of the lytic activity of vB_VspM_VS1 for *V. splendidus*, shown that the infection of phage vB_VspM_VS1 with MOI=0.01 (b) can cleave the entire pathogen on the plate, as a comparison, there were some bacteria can still grow on the plate with phage titer of 0.001 (a), indicating that the isolated phage has extremely strong lytic activity.


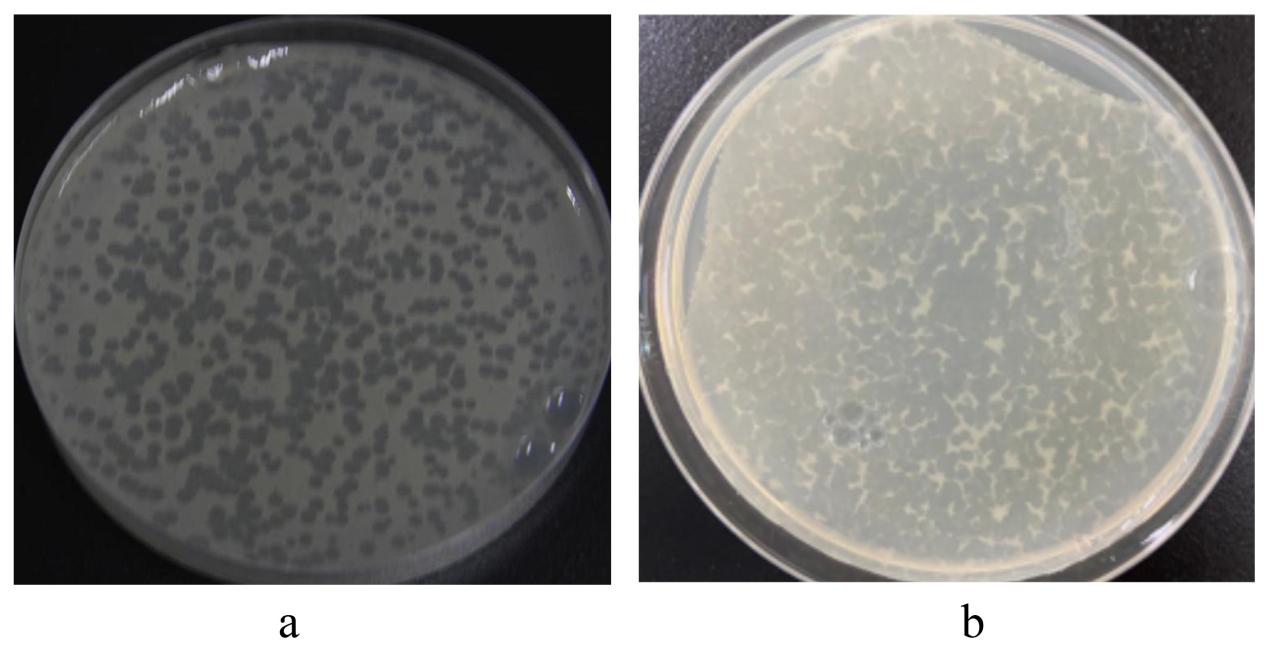

Supplement: S1 File — S1 Fig, Plot of comparative analysis of genome similarity between phage vB_VspM_VS1 and phage nt-1 (HQ317393.2). S2 Fig, The display diagram of the lytic activity of vB_VspM_VS1 for V. splendidus, shown that the infection of phage vB_VspM_VS1 with MOI = 0.01 (b) can cleave the entire pathogen on the plate, as a comparison, there were some bacteria can still grow on the plate with phage titer of 0.001 (a), indicating that the isolated phage has extremely strong lytic activity. (DOC) [file pone.0289895.s001.doc]
